# Characterization of the immune related lncRNAs in bladder cancer to aid immunotherapy

**DOI:** 10.3389/fimmu.2022.941189

**Published:** 2022-08-26

**Authors:** Ying Xiao, Yipeng Dong, Tiannan Yu, Rujie Wang, Yang Gao, Song Li, Shaojun Nong, Wenguang Li

**Affiliations:** ^1^ Department of Urological Surgery, Affiliated Hospital of Nantong University, Nantong, China; ^2^ School of Medicine, Nantong University, Nantong, China; ^3^ Research Center of Clinical Medicine, Affiliated Hospital of Nantong University, Nantong, China; ^4^ Department of Burns and Plastic Surgery, Affiliated Hospital of Nantong University, Nantong, China

**Keywords:** bladder cancer, lncRNAs, TME subtypes, immunotherapy, anti-tumor activity

## Abstract

Bladder cancer (BLCA) is the 10th most common form of cancer worldwide. Currently, the response rate of BLCA patients to novel immunotherapy and immune checkpoint inhibitor (ICI) treatment is around 30% or less. Therefore, there is an urgent clinical demand to understand the regulation of immune function in BLCA patients. LncRNAs are known to play fundamental roles in the regulation of the immune system in the tumor microenvironment. In this report, we performed a comprehensive analysis to identify immune-related lncRNAs (IRLs) in BLCA patients using The Cancer Genome Atlas (TCGA) databases. BLCA patients were divided into five TME subtypes. Subtype HMIE was strongly related to survival and high anti-tumor activity of patients. Through a four-step analysis, we identified 34 IRLs as subtype HMIE related lncRNAs (HMIE-lncs).The correlation analysis with immune cell infiltration and target gene pathway enrichment showed that 34 HMIE-lncs were correlated with immune cell activation and tumor cell killing. Among them, 24 lncRNAs were related to good prognosis. We constructed a risk model to predict BLCA. Cross tumor validation was performed, and the results showed that the 34 HMIE-lncs identified in the BLCA patients in this study were highly expressed in the immune-favorable TME subtype (IE) in most of the other cancer types.

## Graphical Abstract

According to previous reports of four subtypes of tumor patients, we further divided subtype IE into two subtypes of HMIE and LMIE in bladder cancer. Further analysis through four steps identified 34 immune-related lncRNAs (IRLs) in the HMIE subtype. Of these, 24 lncRNAs are related to good prognosis. Following validation, a risk model was constructed to predict BLCA. Cross tumor type studies confirmed that 34 HMIE-lncs were highly expressed in immune-favorable TME subtype (IE) in most of cancers, and Imvigor210 immunotherapy cohort were used to validate these lncRNAs.
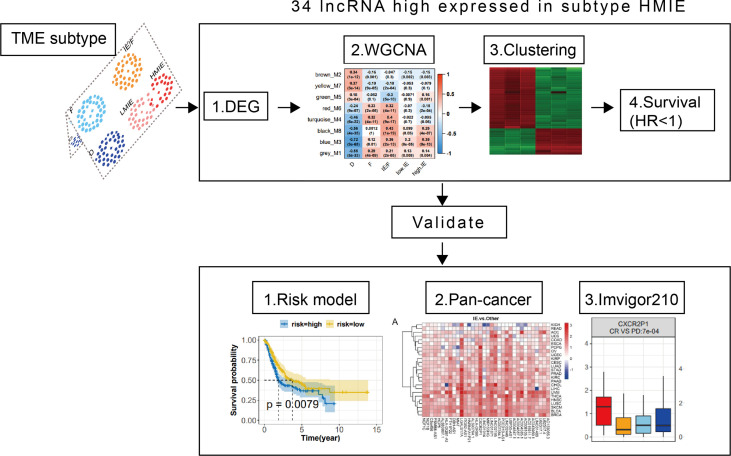

## Introduction

Bladder cancer (BLCA) is a urinary tract malignancy and it ranks as the 10th most common form of cancer worldwide (1). About 70%–80% of newly diagnosed BLCA is non-muscular invasive carcinoma (NMIBC). NMIBC is usually treated with transurethral lumpectomy, a minimally invasive surgical procedure. If the disease does not progress to muscle-invasive carcinoma (MIBC), patients with NMIBC have a good prognosis, and approximately 30% of patients eventually develop MIBC. Radical cystectomy combined with pelvic lymph node dissection is the standard treatment option for local MIBC, but about 50% of patients still suffer local recurrence within two years. The 3-year survival rate is less than 50% (2). Despite the establishment of several novel treatment strategies, BLCA remains a medical challenge for effective treatment (3). Dysregulation of the immune system can be a major cause of the development of cancer. Therefore, immunotherapy has emerged as a promising cancer treatment strategy (4). However, the response rate of BLCA patients to novel immunotherapy and immune checkpoint inhibitor (ICI) treatments is around 30% or less (5). A growing number of genomic analyses suggest that genomic/epigenetic changes in tumor tissue may play an important role in tumor immune response and checkpoint blocking effects (6, 7). However, most previous genotype-immunophenotypic association studies have overlooked the potential impact of lncRNAs on the tumor immune microenvironment, which has become an important regulator of tumorigenesis (8). Thus, there is an urgent clinical demand to understand the regulation of immune responses in BLCA patients. Molecular profiling for BLCA is one of the best research strategies to understand the molecules and signaling pathways regulating the immune response in the BLCA tumor microenvironment. The discovery of important immune molecular signals in the BLCA tumor microenvironment could offer new strategies to manipulate these signaling pathways in order to suppress tumor progression, recurrence, and metastasis (9, 10).

So far, most studies that have performed molecular profiling for BLCA have focused on coding genes, particularly the function of cell-surface receptors, cytokines, and transcription factors. Increasing evidence indicates that lncRNAs play fundamental roles in controlling the function of the immune system (10), and lots of reports show that immune-related lncRNAs (IRLs) play a significant role in the establishment of the tumor microenvironment (TME) (11). For instance, the lncRNA NeST has been found to be a critical regulator of immune response through the activation of T-cells (12). The lncRNA NRON maintains the resting state of T cells by sequestering phosphorylated NFAT in the cytoplasm (13). In contrast, the oncogenic lncRNA LINK-A downregulates cancer cell antigens and intrinsic tumor suppressors (14). In this study, we identified several IRLs in bladder cancer that positively correlated with https://cn.bing.com/search?q=Cytotoxicity&filters=sid%3adbae80d2-b094-bb8c-40c4-19ee13eae6f4&form=ENTLNK lymphocyte cell infiltration and activation. We found that the target genes of these IRLs were involved in T-cell activation, cell killing, and NK cell activity related biological processes. A risk model composed of six IRLs was constructed, and it shows a strong predicted ability in overall survival (OS), and patients with a low risk score have a high anti-tumor signature score. These IRLs were also highly correlated with immune cell infiltration, especially https://cn.bing.com/search?q=Cytotoxicity&filters=sid%3adbae80d2-b094-bb8c-40c4-19ee13eae6f4&form=ENTLNK lymphocyte infiltration across other cancers. Furthermore, several IRLs were found to prolong the survival of patients in the IMvigor210 immunotherapy cohort and have elevated expression in the complete response patient group. They also showed a strong positive correlation with https://cn.bing.com/search?q=Cytotoxicity&filters=sid%3adbae80d2-b094-bb8c-40c4-19ee13eae6f4&form=ENTLNK lymphocyte cell infiltration. In summary, we identified several lncRNAs that are involved in tumor-fighting lymphocyte activation and infiltration, and they could be considered potential biomarkers or important targets for BLCA immunotherapy.

## Methods

### Data sets

Bladder cancer (BLCA) and pan-cancer datasets were obtained from The Cancer Genome Atlas (TCGA). Level 3 RNA-Seq data consisting of 406 BLCA tissues and 18 healthy controls were downloaded from the UCSC Xena browser (https://xena.ucsc.edu/) (15). Non-primary tumors and formalin-fixed paraffin-embedded samples were filtered out, and then one sample from each patient was selected. A total of 403 BLCA samples were finally included in this study. Corresponding clinical characteristics, therapeutic regimen, corresponding response, follow-up, RNA-Seq, and somatic mutation data were obtained. The details of clinical–pathological characteristics for the TCGA-BLCA cohort are summarized in **Table S1**. Twenty-five other TCGA solid tumors were used in this study. Transcriptome and clinical data were also collected from the UCSC Xena browser. One immunotherapy cohort (IMvigor 210) was downloaded from http://research-pub.gene.com/IMvigor210CoreBiologies. Data on RNA-seq were transcripts per million (TPM) normalized and log2-transformed. Then, genes with low expression were eliminated. The tumor mutation burden (TMB) was defined as the total number of nonsynonymous alterations (SNVs or indels) for each patient.

### TME subtypes

Patients were first divided into four subtypes (D, F, IE/F, and IE) according to a previous study (16). Following analysis, we further divided the IE subtype into two subtypes as low the TMB score subtype and the high TMB score subtype, separated by the median value of TMB score.

### Detection of differential expression molecules between groups

For the RNA-seq data, edgeR and DEseq2 R packages were used. Molecules with a FDR <0.05 and an absolute fold change of >1.5 were considered as differentially expressed.

### Functional characterization of target genes

The Kyoto Encyclopedia of Genes and Genomes (KEGG) database and Gene Ontology (GO) category database were used for functional annotation of the target genes of lncRNA. Only those GO categories or pathways containing at least five DEGs were kept for further analysis. The enrichment analysis of GO categories was performed by the R cluster Profiler (v3.14.3) package (17), and the enrichment analysis of pathways was tested upon a hypergeometric distribution by the R ‘phyper’ function. Those GO categories with a false discovery rate (FDR) of <0.05 were considered significantly enriched, while pathways with a p-value of <0.05 were enriched.

### Weighted gene co-expression network analysis (WGCNA)

WGCNA was performed by the R package WGCNA (V1.69) (18). We used the log2 transformed TPM value as the normalized expression and detected outlier samples by the ‘hclust’ function. According to the principle of scale-free networks, the weighting coefficient β was determined as 9 using the integrated function (pickSoft Threshold) in the WGCNA package. The network type was set as ‘signed’ and ‘bicor’ (bi-weighted correlation) was used to calculate the correlation adjacency matrix. The co-expressed gene modules were identified using a dynamic tree cut with the following major parameters: minModule Size of 30 and deep Split of 1. Some highly similar modules with the height of module eigengene in the clustering lower than 0.2 were merged.

### Transcriptomic signatures

The abundance of infiltrating immune cell populations was measured using the MCP-counter (19). The immune score, tumor purity, and stromal score for tumor samples were calculated using the R package “ESTIMATE” (5). Other immune or tumor-associated signatures in each sample were calculated using the ssGSEA. Pathway activity scores (N = 11) were calculated using PROGENy (20). Supplement: The immunologic signatures were downloaded from the Immport database (21).

### Risk-score model

Univariate Cox regression, LASSO regression, and stepwise regression were used in sequence to single out candidates for lncRNA in the model. lncRNAs that met the criteria of p-value <0.05 were considered as survival related to univariate Cox proportional hazard regression analysis. The criteria of the LASSO regression were to be retained in the model more than 950 times in all 1,000 repetitions. Then, the stepwise method was used. The risk-score model was constructed based on Cox regression coefficients and the expression of lncRNAs. Risk score = β1X1 + β2X2 +……+ βmXm, in which β indicates the regression coefficients for each gene and X indicates the gene expression profile. K–M survival analysis and ROC curves were performed to evaluate the predictive accuracy of models.

### Searched for lncRNA cis-regulated target genes

The putative cis-acting regulatory DNA elements (cis-elements) regulate the transcription of neighboring genes. With the help of gene chromosome coordinates, this study defined genes located within 10 kbp upstream or downstream of the lncRNAs to be cis-acting target genes.

### Statistical analysis

Hierarchical clustering analysis was performed on the R ‘hclust’ function using the ‘ward.D’ method to identify the number of clusters in the TCGA-BLCA cohort based on the expression pattern of lncRNAs. The Kaplan–Meier method and log-rank test were conducted to compare survival differences between the two tumor groups. The optimal cutoff point in the expression of each lncRNA was determined using the R package “survminer.” Then, the cutoff values determined were used for categorizing groups. Prediction of OS was performed using univariate Cox proportional regression analysis. Univariate and multivariate Cox proportional hazards regression models were used to assess the association between the clinical factors and the independence associated with prognosis. The hazard ratio (HR) and 95% confidence interval (CI) were calculated. One-tailed or two-tailed Wilcoxon rank-sum or student tests were used to compare the two groups. For comparisons of more than two groups, the one-way ANOVA test and the Kruskal–Wallis test were used as parametric and nonparametric methods, respectively. If unnoted, there is no statistical significance in a one-by-one comparison. All statistical analyses were performed using the R/Bioconductor (version 3.6.1).

## Results

### Define five TME subtypes in TCGA-BLCA cohort

As reported in previous studies, tumor patients could be broadly classified into four subtypes based on the expression of functional gene signatures (16). The four subtypes include (1) immune-enriched, non-fibrotic (IE) subtype, (2) immune-enriched, fibrotic (IE/F) subtype, (3) fibrotic (F) subtype, and (4) immune-depleted (D) subtype. These subtypes were conserved across 20 different cancers, and these subtypes are correlated with the response of the patient to immunotherapy. Our analysis revealed that, in bladder cancer, subtype IE rather than subtype IE/F melanomas are enriched with an immune-inflamed histological phenotype characterized by abundant lymphocyte infiltration. Subtype IE also showed a strong positive correlation with the survival of the patient (**Figure S1A**). High tumor mutation burden (TMB) has been considered a promising immune-response biomarker and TMB correlates with immune checkpoint blockade response (22). However, TMB could not be used to differentiate a potential responder from a non-responder (AUC = 0.56). Therefore, TMB prediction shows only a 50% chance of correctly predicting the response. Whereas incorporating the TME classification before and during the treatment increased the accuracy from 50% to 80% (16).

**Figure 1:**
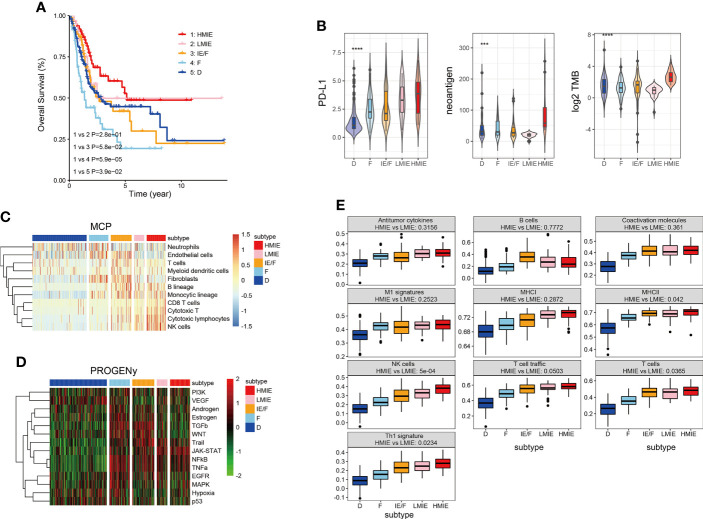
Five distinct TME subtypes identified in bladder cancer. **(A)** Overall survival of bladder cancer patients is stratified by TME subtype classification in the TCGA-BLCA cohort. The P-value was determined by log-rank. **(B)** Violin plots showing the difference in PD-L1expression, neoantigen load and log2 transformed total mutational burden across the five TME subtypes. Statistical significance was calculated by the one-way ANOVA test. **(C)** Heatmap of cell type specific gene signature scores retrieved from the MCP-counter cell deconvolution algorithm. Samples in the column were grouped according to TME subtypes and cell types in the row were clustered by the ‘Ward’ algorithm. **(D)** Heatmap of relative signaling pathway activity scores by PROGENy. Samples in the column were grouped according to TME subtypes and pathway activity scores in the row were clustered by the ‘Ward’ algorithm. **(E)** Boxplot of 10 gene signature scores across five TME subtypes measured by ssGSEA algorithm. Statistical significance was calculated by the one-way ANOVA test. These gene signatures were defined as anti-tumor microenvironments (11). ***p<0.001 ****p<0.0001.

In this study, we first incorporated the TMB score and TME classification to analyze the TME subtypes of the TCGA-BLCA cohort. Our analysis revealed that TMB is high in subtype IE and subtype D, with subtype IE showing the highest TMB score (**Figure S1C**). TMB was statistically higher in subtype IE than in the non-IE subtype (**Figure S1C**). Next, we tested whether an IE or non-IE subtype with high or low TMB has a different survival effect. Patients were divided into four subtypes: IE subtype with high TMB, IE subtype with low TMB, non-IE subtype with high TMB, and non-IE subtype with low TMB. Further analysis showed that the IE subtype with high TMB is positively correlated with survival, but the IE subtype with low TMB had a similar effect to the non-IE subtype with high TMB on patient survival (**Figure S1B**). This finding suggests that it is important to incorporate both TMB and TME to predict patient survival. Therefore, we further divided the subtype IE into two separate subtypes: an IE subtype with high TMB (HMIE), and an IE subtype with low TMB (LMIE). Although stromal score and immune score did not differ significantly between subtypes HMIE and LMIE (**Figure S2**), patients with subtype HMIE had longer overall survival (**Figure 1A**). Therefore, we used five subtypes (HMIE, LMIE, IE/F, F, and D) for the following analysis.

Following the subtype classification, we tested whether patients in the HMIE subtype showed a high level of tumor-fighting lymphocyte infiltration and anti-tumor activity. Previous studies have explored potential biomarkers to predict patient response to https://www.sciencedirect.com/science/article/pii/S0091674918304457 (ICB) treatment, these biomarkers include the expression of checkpoints PD-L1 (23), the tumor mutation burden (TMB) (24), and neoantigen load (25). PD-L1 expression was gradually increased from subtype D to subtype IEF and then to subtype HMIE (**Figure 1B**). Neoantigens trigger immune responses and modulate immune infiltration in the tumor microenvironment (TME). It often leads to those tumors being more sensitive to ICB and resulting in better prognosis (26, 27). The HMIE subtype was enriched with neoantigens (**Figure 1B**). The heatmap of immune cell infiltration inferred using the MCP-counter cell deconvolution method showed high infiltration of https://cn.bing.com/search?q=Cytotoxicity&filters=sid%3adbae80d2-b094-bb8c-40c4-19ee13eae6f4&form=ENTLNK T, CD8 T, and NK cells in the HMIE subtype (**Figure 1C**). PROGENy algorithm analysis (20) revealed that immune activation related pathways such as NF-kB, JAK-STAT, and TRAIL signaling pathways were elevated in the HMIE subtype (**Figure 1D**). Next, we studied the activity of the anti-tumor gene signature across five subtypes, and tested the statistical difference between HMIE and LMIE subtypes (**Figure 1E**). All these anti-tumor signatures were increased gradually across the five subtypes from subtype D to HMIE. Five gene sets, including MHCII antigen, NK cell, T-cell infiltration, Th1 signatures, and TLS (tertiary lymphoid structures), were statistically higher in subtype HMIE than in LMIE. NK cells showed the most significant difference (p-value = 5e−4). Altogether, our analysis by incorporating the TMB score and TME classification to classify the subtypes in the TCGA-BLCA cohort identified an HMIE subtype showing hot tumor characteristics, showing long survival, high level of neoantigen, cytoxic T, and NK infiltration and activities. Thus, the HMIE subtype may have a stronger response to novel immunotherapy and immune checkpoint blockade (ICB) treatment.

### Identify 34 HMIE subtype specific lncRNAs in the TCGA-BLCA cohort

**Figure 2:**
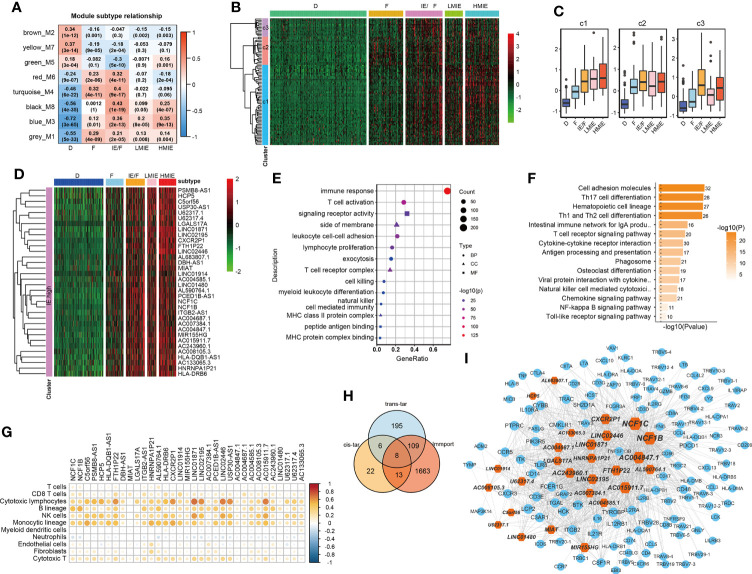
Identification of HMIE subtype specific lncRNAs. **(A)** Correlation between https://www.rdocumentation.org/packages/WGCNA/versions/1.70-3/topics/moduleEigengenes (first principal component of modules) and TME subtypes on TCGA-BLCA cohort. The correlation was shown as a heatmap which was gradually colored with lower in blue and higher in red according to the Pearson correlation coefficient. The first line of the values in the heatmap represents the correlation coefficient, and the second line are the p-values from the correlation test. Genes that belong to M3 and M8 modules were identified with high expression in immune enriched subtypes of IE/F, LMIE, and HMIE. **(B)** Gene expression clustering of lncRNA assigned to M3 and M8 modules. lncRNAs were clustered into three groups. The heatmap showed the expression profiles of these three groups of lncRNAs, which are colored with lower in green and higher in red according to the expression of the lncRNA. **(C)** A boxplot showing the difference in expression across the three groups of lncRNAs. **(D)** A heatmap of 34 lncRNAsthat are highly expressed in the HMIE subtype, and they were defined as HMIE-lncs. **(E)** GO enrichment of trans-targets that are co-expressed with 34 HMIE-lncs. **(F)** KEGG enrichment of trans-targets that are co-expressed with 34 HMIE-lncs. **(G)** Correlation analysis between 34 HMIE-lncs expression and immune cell infiltration. The positive correlation coefficient was shown in orange and the negative correlation coefficient was shown in blue. Only pairs having a p-value of <0.05 with a correlation test were plotted. **(H)** Venn plot of three gene sets: trans-target of and cis-targets of the 34 HMIE-lnc, and genes annotated from Immport database. **(I)** Network plot of HMIE-lncs and its immune related targets. lncRNAs were shown in hexagon shapes and colored in orange; target genes were shown in circle shapes and colored in skyblue. All nodes in the network were sized gradually by the value of outdegree.

As reported, lncRNAs can play fundamental roles in the regulation of the immune system (10). Next, we aimed to identify lncRNAs that regulate immune cell infiltration and identify lncRNAs that have anti-tumor activities in bladder cancer. As discovered in the above analysis, the HMIE subtype presents hot tumor characters with a high level of tumor-fighting lymphocyte infiltration and anti-tumor activity. We therefore screened the expression of lncRNAs in the HMIE subtype through four steps of analysis, and the analysis identified 34 lncRNAs that are highly expressed in subtype HMIE. In the first step, we examined differentially expressed RNA, including both lncRNA and mRNA, in each subtype and identified 2,842 subtype-specific lncRNAs and 5,729 subtype-specific mRNAs. In the second step of WGCNA analysis, subtype-specific RNAs with low expression were filtered out to keep lncRNAs with ≥15% expression and mRNAs with ≥20% expression. With these criteria of filtration, 1,877 lncRNAs and 4,311 mRNAs were kept for WGCNA analysis. One outlier sample was further removed by hierarchical clustering. In our study, a power of β = 9 (scale freeR2 = 0.88) as the soft threshold was adopted to achieve a scale free network (**Figure S3A**). As shown in **Figure S3B**, seven co-expressed genes were identified using the ‘cutreeHybrid’ function (**Figure S3B**). Genes in the black and blue modules were highly expressed in immune cell infiltrated subtypes such as IE/F, LMIE, and HMIE subtypes (**Figure 2A**). This indicates that genes in the black and blue module (759 molecules) may be responsible for immune cell infiltration. KEGG pathway enrichment revealed that genes in the blue module were important regulators for immune system related pathways such as cytokine–cytokine interaction, antigen processing and presentation, and Th17 cell differentiation (**Figure S2C**). However, we did not find a module with an increased positive correlation with the HMIE subtype. We therefore focused on lncRNA analysis in the third step. We combined lncRNAs in both black and blue modules and grouped them into three clusters according to the expression profile of these lncRNAs by hierarchical clustering (**Figure 2B**). Interestingly, cluster 1 lncRNAs have elevated expression in the HMIE subtype (**Figure 2C**). Given that the HMIE subtype is associated with good prognosis, cluster 1 lncRNAs could be associated with good prognosis as well. In the last step, we calculated the hazard ratio (HR) value of lncRNAs by stratifying the univariate Cox proportional hazards regression model. LncRNAs in cluster 1 with a HR value <1 were considered as HMIE specific lncRNAs for good prognosis. Through the above four-step analysis, we identified 34 HMIE-specific lncRNAs in the TCGA-BLCA cohort. The heatmap of identified 34 lncRNAs showed that these lncRNAs were highly expressed in the HMIE subtype (**Figure 2D**).

Several lncRNAs could co-express in the same cell to regulate their target, which was named trans-target (28). We correlated trans-targets of identified 34 lncRNAs from the black and blue modules by the ‘export Network To Cytoscape’ function of WGCNA. These trans-targets showed marked enrichment of immune activation-related biological processes such as T-cell activation, lymphocyte proliferation, cell killing, NK cell immunity, and MHCI and MHCII complex activity (**Figure 2E**). The KEGG pathway enrichment analysis also showed a significant enrichment of immune-related of pathways (**Figure 2F**). By correlation analysis with immune cell infiltration, we found these 34 lncRNAs were indeed positively correlated with B, cytotoxic T cell, NK, and monocyte-lineage cell infiltration (**Figure 2G**). In addition to trans-targets, lncRNA can regulate the expression of neighboring genes, which are known as cis-targets. We then filtered out both cis-targets and trans-targets for these lncRNAs from ImmPort (21), one of the largest open repositories of immunological data, to identify genes having functions in the immune system. Among 308 identified trans-targets, 115 genes (109 + 8 + 13) show intersection with genes annotated by Immport (37%), and nearly 34% of cis-target genes have Immport annotation (**Figure 2H**). These cis-targets and trans-targets annotated by Immport were therefore regarded as immune activation-related targets for lncRNAs in the HMIE subtype. The interaction between the 34 HMIE subtype-specific lncRNAs and their immune activation-related targets was plotted as a network by Cytoscape 3.6 software. The plot comprises 150 nodes, including 27 lncRNAs and 123 target genes. These nodes formed 1,074 network pairs (**Figure 2I**). Several lncRNAs such as NCF1C, NCF1B, CXCR2P1, LINC02446, and AC0048473.1 have a high number of target genes that are associated with immune function, indicating that these lncRNAs are functionally related to immune activation in bladder cancer.

### Construct a risk model as a predictor of prognosis in BLCA patients

**Figure 3:**
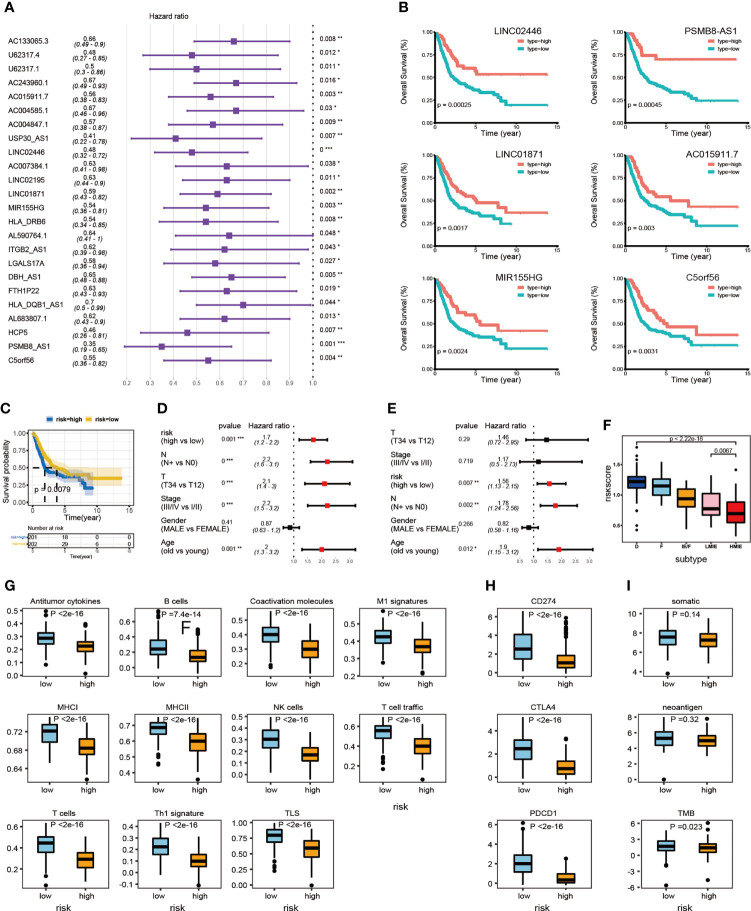
LncRNA expression and clinical prognosis. **(A)** Result of HMIE-lncs by univariate Cox regression analysis. HR and p-values were displayed. Only the lncRNAs with a p-value of <0.05 were plotted. All plotted lncRNA have an HR value <1. **(B)** The Kaplan–Meier survival curve of six lncRNAs that are significantly related to the survival of bladder cancer. Log-rank p-values were shown. Higher expression was related to a good prognosis. **(C)** The Kaplan–Meier curves compared patients with low or high immune risk in the TCGA-BLCA cohort. Patients were divided into two groups according to the median value of lncRNA risk scores. Higher risk scores were correlated with poorer prognosis. **(D)** Univariate COX regression analysis of clinical factors. **(E)** Multivariate Cox regression analysis of clinical factors. **(F)** The distribution of lncRNA risk score across five TME subtypes. The risk score is low in the HMIE subtype and high in both the D and F subtypes. **(G)** The distribution of anti-tumor scores between high and low-risk groups. **(H)** The distribution of expression of three immune checkpoint molecules between high and low-risk groups was determined. **(I)** The distribution of somatic mutation, neoantigen load and log2-transformed TMB score between high and low-risk groups. *p<0.05, **p<0.01, ***p<0.001, ****p<0.0001.

We further studied the correlation between the expression of identified 34 lncRNAs in the HMIE subtype and survival of patient in the TCGA-BLCA cohort. The optimal cutoff point for the expression of each lncRNA was determined by the R package “survminer” and then a stratified univariate Cox proportional hazard regression analysis was performed. Following the analysis, 24 lncRNAs were found to be associated with a good prognosis with p-value <0.05 and a hazard ratio (HR) value <1 (**Figure 3A**). Six lncRNAs, LINC02446, PSMB8-AS1, LINC01871, C5orf56, MIR155HG, and AC015911.7 were more significantly related to the survival of patients (p-value <0.005). The K–M survival curve for these six lncRNAs was plotted (**Figure 3B**). Previous reports showed that LINC02446 could affect the proliferation, migration, and invasion of bladder cancer cells (29) while LINC01871 has the effect of prolonging the survival time of bladder cancer patients (30). To construct the lncRNA risk model, we performed the least absolute shrinkage and selector operation (LASSO) regression analysis, and eight lncRNAs were included more than 950 times in all 1,000 repetitions for the analysis. Following stepwise regression, a model based on the expression of five lncRNAs in the TCGA-BLCA cohort was constructed: lncRNA risk score = AL683807.1 ∗ (−0.189741689) + LINC02446 ∗ (−0.141850798) + PSMB8-AS1 ∗ (0.035933025) + U62317.4 ∗ (0.116368232) + USP30-AS1 ∗ (−0.089116129). Based on the median risk score, the total TCGA-BLCA set was divided into high- and low-risk groups, respectively. K–M analysis and receiver operating characteristic (ROC) curves were performed to validate the predictive ability of the lncRNA risk model. Patients in the high-risk groups have a worse prognosis than the low-risk groups (log-rank test, p = 0.0079, **Figure 3C**). According to the ROC curves of 1-, 3-, and 5-year OS predictions, the area under the curve (AUC) was above 0.6 (**Figure S4**). The value of HR was 1.68 (1.25–2.26, p-value = 0.001) in the high risk group by univariate Cox regression, which showed that our risk model could be an independent predictor of prognosis in BLCA patients. The value of HR was 1.68 (1.25–2.26, pvalue = 0.001) and 1.68 (1.13–2.15, pvalue = 0.007) in the high risk group by univariate and multivariate Cox regression separately, which showed that our risk model could be an independent predictor of prognosis in BLCA patient (**Figures 3D, E**).

Subsequently, we investigated the correlation between the lncRNA risk model and immune cell infiltration and activation in bladder cancer. The risk score was low in the immune infiltration subtype, especially in the HMIE subtype. The risk score in the HMIE subtype was significantly lower in the HMIE subtype than that in the LMIE subtype (p-value = 0.0067, **Figure 3F**). In contrast, subtype F and subtype D exhibited higher risk scores (**Figure 3F**). The boxplot for anti-tumor signature activity revealed that patients with a low-risk score show significant high anti-tumor activity (the maximum p-value<1e−13; (**Figure 3G**). We also found that three important immune inhibitory checkpoints, PDL1 (CD274), CTLA4, and PD1 (PDCD1), were elevated in the low-risk group (**Figure 3H**). There were also high somatic muation, neoantigen load and TMB score in the low-risk group (**Figure 3I**) Thus, our analysis indicated that the lncRNA risk model could be used as a predictor of prognosis that is related to the immune cell infiltration microenvironment in BLCA patients.

### Validation the association of HMIE-related lncRNAs with prognosis in pan-cancer

**Figure 4:**
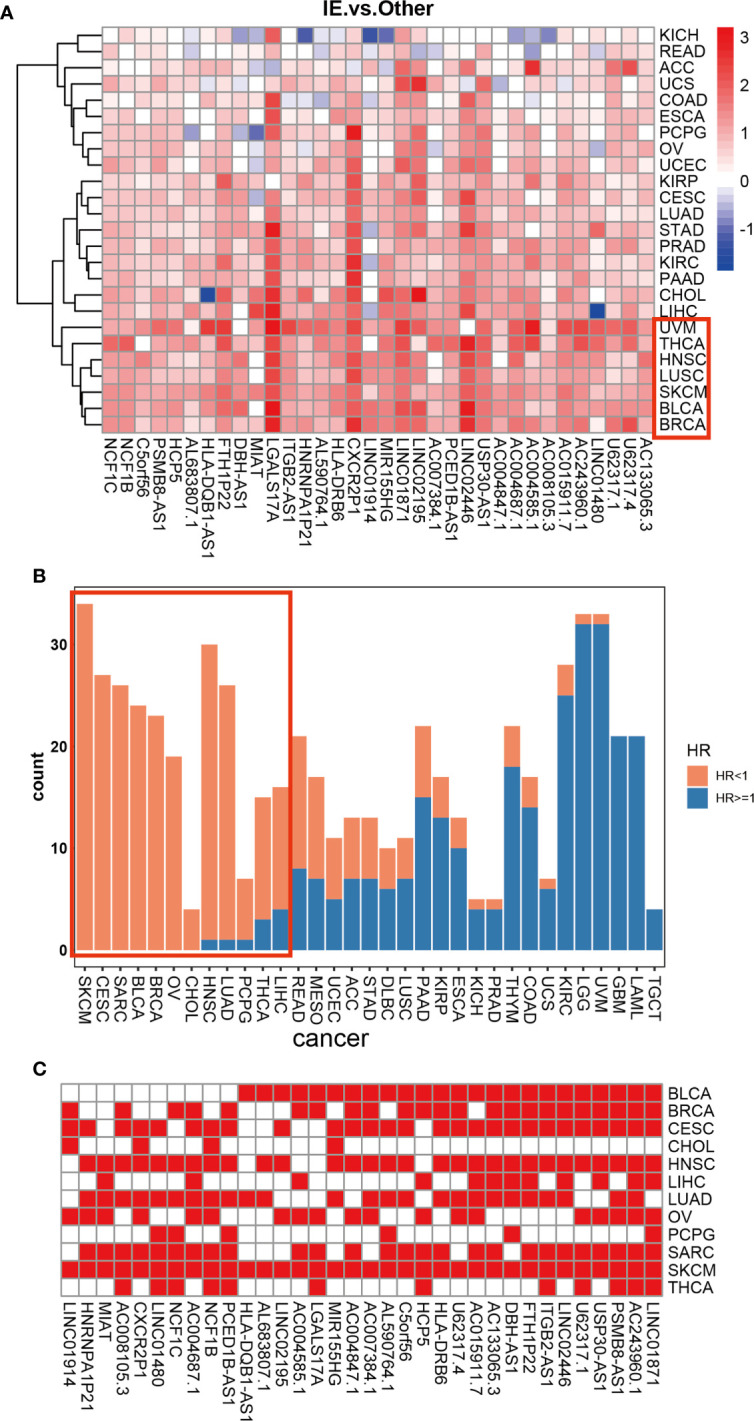
Differential expression of 34 HMIE-lncs in 25 solid tumors. **(A)** The differential expression of 34 HMIE-lncs in 25 solid tumors. The heatmap plot showed the value of log2-foldchange between IE and other TME subtypes for each lncRNA. Log2-foldchange <0 was colored in blue, while log2-foldchange >0 were colored in red. The dark color of the red or blue indicates higher log2-Foldchanges. **(B)** Barplot shows the number of lncRNAs that met the requirements of p-value <0.05 in each cancer from the TCGA database. Bars were colored with an HR ratio <1 (orange) or ≥1 (blue). Twelve cancers containing more than 66% of lncRNAs with HR <1 were highlighted in the red box. **(C)** Heatmap showing lncRNAs that are good prognosis related across 12 cancers.

Given our findings of 34 HMIE-related lncRNAs in bladder cancer, we studied if HMIE-related lncRNAs are bladder cancer-specific or if they are common features of other types of cancer. We compared the expression of 34 HMIE-related lncRNAs between the IE subtype and other subtypes in 25 TCGA solid tumor cohorts, including BLCA. Patients in each cohort were divided into four subtypes according to Alexander et al. (16). We calculated their expression by the log2 transformed fold-change for the identified 34 lncRNAs between the IE subtype and other non-IE subtypes and plotted them in a heatmap (**Figure 4A**). Twenty-five solid tumor cancers were then clustered by hierarchical clustering based on log2 transformed fold-changes. The majority of the cancers, except kidney chromophobe (KICH) and rectum adenocarcinoma esophageal carcinoma (READ), show upregulation of these lncRNAs in the IE subtype compared with non-IE subtypes. The cancers highlighted in the red box in **Figure 4A** were the most significant cancer types that have an upregulation of these lncRNAs. The optimal cutoff point for the expression of each lncRNA was determined and then stratified univariate Cox proportional hazard regression analysis was performed for each cancer type. LncRNAs having a p-value of <0.05 were regarded as survival-related, and the number of survival-related lncRNAs with HR >1 or HR <1 was counted and plotted with a bar-plot. Twelve cancers were highlighted in the red box in **Figure 4B** that have more than 66% lncRNA upregulation with HR <1 (**Figure 4B**). LncRNAs with good prognosis across these 12 cancers are plotted in a heatmap (**Figure 4C**). Twenty-four lncRNAs discovered to be survival related in bladder cancer with a favorable prognosis are also appropriate in at least one other type of cancer.

### Validation of identified 34 HMIE-lncs in immunotherapy dataset IMvigor 210 cohort

**Figure 5:**
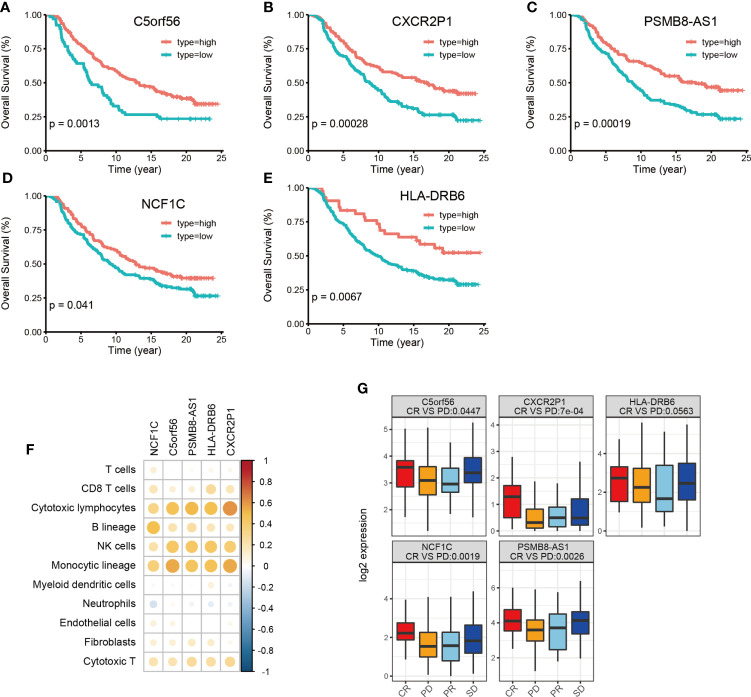
Validation of identified 34 HMIE-lncs in immunotherapy dataset of the IMvigor 210 cohort. **(A–E)** Overall survival of 5lncRNAs that met log-rank p-value <0.05 in PD-L1 treated immunotherapy dataset of the IMvigor 210 cohort. **(F)** Correlation analysis between five survival related lncRNA expression and immune cell infiltration. Positive correlation coefficients are shown in orange and negative correlation coefficients are shown in blue. The darker color indicates a bigger value. Only the pairs with a correlation test p-value <0.05 were plotted. **(G)** Boxplot showing the difference of five lncRNA expression across four therapy response group. CR represents complete response, PD represents progressive disease, PR represents partial response, and SD represents steady disease.

Finally, we used the immunotherapy IMvigor 210 cohort and studied the association of lncRNA with immunotherapy (31). A total of 167 lncRNAs were considered to have an expression in the IMvigor 210 cohort with a criterion that at least 10% of the samples have the expression and a TPM value >1. However, we identified 16028 lncRNAs in the TCGA-BLCA cohort with the same criterion. Among the 34 HMIE-lncs identified in the TCGA-BLCA cohort, six lncRNAs were expressed in the IMvigor 210 cohort. Among these expressed lncRNAs, five were associated with good prognosis (log-rank p-value <0.05, **Figures 5A–E**). These five lncRNAs were positively correlated with B, cytotoxic T cell, NK, and DC cell infiltration (**Figure 5F**), and all of them had a significantly higher expression in the complete response group than in the progressive disease group. CXCR2P1 had the most significant value (**Figure 5G**). In addition, PSMB8-AS1, C5orf56, and HLA-DRB6 were found in the TCGA-BLCA dataset that they were significantly correlated with survival (p-value <0.01, **Figure 3B**). The other two lncRNAs, CXCR2P1 and NCF1C, may play an important role in the regulation of immune gene expression because they both show relatively high out-degree from the lncRNA and target network (**Figure 2I**). Due to the small amount of lncRNA expression in the IMvigor 210 cohort, we were unable to fully validate the effect of the 34 lncRNAs in the IMvigor 210 cohort. However, this validation study indicates that these lncRNAs are good prognosis-related lncRNAs because five of six lncRNAs in the IMvigor 210 cohort were good prognosis-related lncRNAs.

## Discussion

The human immune system has the function of monitoring and eliminating tumor cells. However, tumor cells have the ability to evade the monitoring of the immune system through immune escape and immunosuppression. Abnormal immune responses are closely related to the occurrence and development of tumor (32). In recent years, studies have found that lncRNA plays an important regulatory role in immune response, including regulating the development, differentiation, and activation of immune cells, and regulation of cytokine receptor regulation of immune response (33).

LncRNAs have known function to regulate cancer immunity and tumor microenvironment in bladder cancer (34). Several immune-related lncRNA models have been constructed according to published literature (35–37). In those studies, lncRNAs were found to be immune related only by co-expression with immune response related genes or by correlation with immune infiltration score, but those models have pitfalls for good prognosis. For example, samples with high immune scores were also shown to coexist with fibroblasts in the IEF subtype (16), but the prognosis of IEF patients is poor. The incidence and clinical characteristics of bladder cancer, such as pathological classification, tissue type, and occurrence site, are significantly different in different patients, and the prognosis of patients at the same stage is also different. Therefore, more knowledge is needed about the prognostic factors of bladder cancer. In this study, we first divided bladder cancer patients into four subtypes: IE, IEF, F, and D (16). Following initial analysis, we further subdivided the IE subtype into HMIE and LMIE subtypes according to the level of TMB value. Patients in the HMIE subtype were found to be more effective on immunotherapy. Patients with the HMIE subtype also have better survival and significantly higher infiltration of NK and T cells, as well as an elevated MHCII antigen level compared with the LMIE subtype. Further investigations through the WGCNA algorithm, hierarchical clustering, and univariate cox regression analysis, we identified 34 lncRNAs that are highly expressed in the HMIE subtype. Through correlation analysis we confirmed that these 34 lncRNAs were significantly correlated with CD8T, cytoxic T, and NK cell infiltration.

In previous studies, statistical methods were used to correlate lncRNA expression with prognosis, but only a few lncRNAs have been identified to construct risk models for good prognosis. Some signatures of immunotherapy, such as immune cell infiltration and the expression of immune checkpoint inhibitors, were used to evaluate the model for immunotherapy prediction. However, those studies did not investigate if the identified lncRNAs were functionally related to immunity. In fact, lncRNAs are more likely to have functions regulating downstream gene expression (38). In this study, the potential target genes were investigated to explore the possible biological functions regulated by immune-related lncRNAs. Functional enrichment analysis revealed that target genes of these lncRNAs were related to T-cell activation, the MHCII complex, cell killing, and NK cell-mediated immunity. We further verified that these lncRNAs are immune response related and are associated with immunotherapy. We then constructed a risk model based on 6lncRNAs for good prognosis. Using this risk model, we show that the anticancer signature was significantly increased in the low-risk group (p-value <1e−16).

Currently, majority of available bladder cancer datasets were performed by microarray analysis, but microarray analysis contains little information about the expression of lncRNA. Therefore, it is difficult to validate our findings in other bladder cancer cohorts. Alternatively, we used a pan-cancer cohort to validate our findings. The HMIE subtype specific lncRNAs in bladder cancer were verified to be highly expressed in the vast majority of other types of cancers in the IE subtype, which is under immune infiltration. Furthermore, our validation results revealed that lncRNAs that are significantly associated with prognosis in bladder cancer are present in several cancer types. This also demonstrates the potential of immune-associated lncRNAs as BC therapeutic targets in bladder cancer patients and may be of great significance in future clinical applications. Moreover, the association of these identified lncRNAs with the prognosis of bladder cancer has been reported in previous publications (25, 26, 29, 30). These reports further support the reliability of our findings. In addition, we used the immunotherapy data of urothelial carcinoma (IMvigor 210 cohort) and validated the predictive value of these lncRNAs in immunotherapy. Although only six of the identified 34 lncRNAs show expression in urothelial carcinoma, five of them are significantly associated with good prognosis. This finding also indicates that this group of genes may become prognostic markers of BC in the future, which could enable clinicians to more conveniently and accurately assess the prognosis of BC patients so as to select more timely and effective immunotherapy for personalized intervention of their conditions, and provide more reliable medical security for patients. The identification of a low number of lncRNAs in urothelial carcinoma could be the reason that the IMvigor210 cohort has a low number of lncRNA expression. Indeed, we only detected 167 lncRNAs in the IMvigor210 cohort. Both CXCR2P1 and NCF1Care are good prognosis-related lncRNAs in the IMvigor210 cohort, and both of them show a function in the regulation of immune-related genes in the TCGA-BLCA, but both of them were not associated with survival. This indicates that identification of lncRNAs only by survival analysis but ignoring their functions may lose some key information.

## Data availability statement

The original contributions presented in the study are included in the article/**Supplementary Material**. Further inquiries can be directed to the corresponding authors.

## Author contributions

Conceived and designed the experiments: WL and SN. Analyzed the data: YX (Investigation, Methodology), YD (Investigation), TY (Methodology), RW (Methodology), YG (Validation), and SL (Validation). Wrote the manuscript: YX and SN. All authors contributed to the article and approved the submitted version.

## Funding

This work was supported by the National Natural Science Foundation of China (Nos. 81802580 and 81771571), the Science and Technology Project of Nantong City (No. JC2018102), and the Postgraduate Research & Practice Innovation Program of Jiangsu Province (No. SJCX21_1460).

## Acknowledgments

We thank the Suzhou Lingdian Biotechnology Co., Ltd. for providing guidance on transcriptome data analysis.

## Conflict of interest

The authors declare that the research was conducted in the absence of any commercial or financial relationships that could be construed as a potential conflict of interest.

## Publisher’s note

All claims expressed in this article are solely those of the authors and do not necessarily represent those of their affiliated organizations, or those of the publisher, the editors and the reviewers. Any product that may be evaluated in this article, or claim that may be made by its manufacturer, is not guaranteed or endorsed by the publisher.

## Supplementary material

Supplementary Figure 1 Survival analysis based on TME subtypes and TMB patient group. **(A)** Overall survival of bladder cancer patients stratified by four TME subtype classification on the TCGA-BLCA cohort. **(B)** Overall survival of bladder cancer patients grouped by the TMB value larger than or smaller than the median of TMB value. Patients with high TMB value have a good survival. **(C)** Boxplot of log2 transformed TMB value across the four TME subtypes. TMB is the highest in IE subtype. **(D)** Boxplot of log2 transformed TMB value across the 2 TME subtypes. Subtypes rather than IE were grouped together as other.

Supplementary Figure 2 Violin plots. **(A)** The distribution of Immune score between 5 TME subtypes, pvalue were determined by ANOVA-test. **(B)** The distribution of Stromal score between 5 TME subtypes, pvalue were determined by ANOVA-test.

Supplementary Figure 3 WGCNA construction. **(A)** Determine soft-thresholding power in WGCNA. **(B)** The scale-free fit index for various soft-thresholding powers (β) (left). The mean connectivity for various soft thresholding powers (right).**(C)**WGCNA cluster dendrogram on bladder cancer patients, genes were grouped into 7 distinct modules.**(D)** KEGG pathway enrichment of genes belong to M3 module.

Supplementary Figure 4 ROC curve for lncRNA risk-model.
