# Supplementary figures and images for: Characterization of the immune related lncRNAs in bladder cancer to aid immunotherapy
Source: Front Immunol. 2022 Aug 26;13:941189. doi: 10.3389/fimmu.2022.941189 (PMC9462669; doi:10.3389/fimmu.2022.941189)

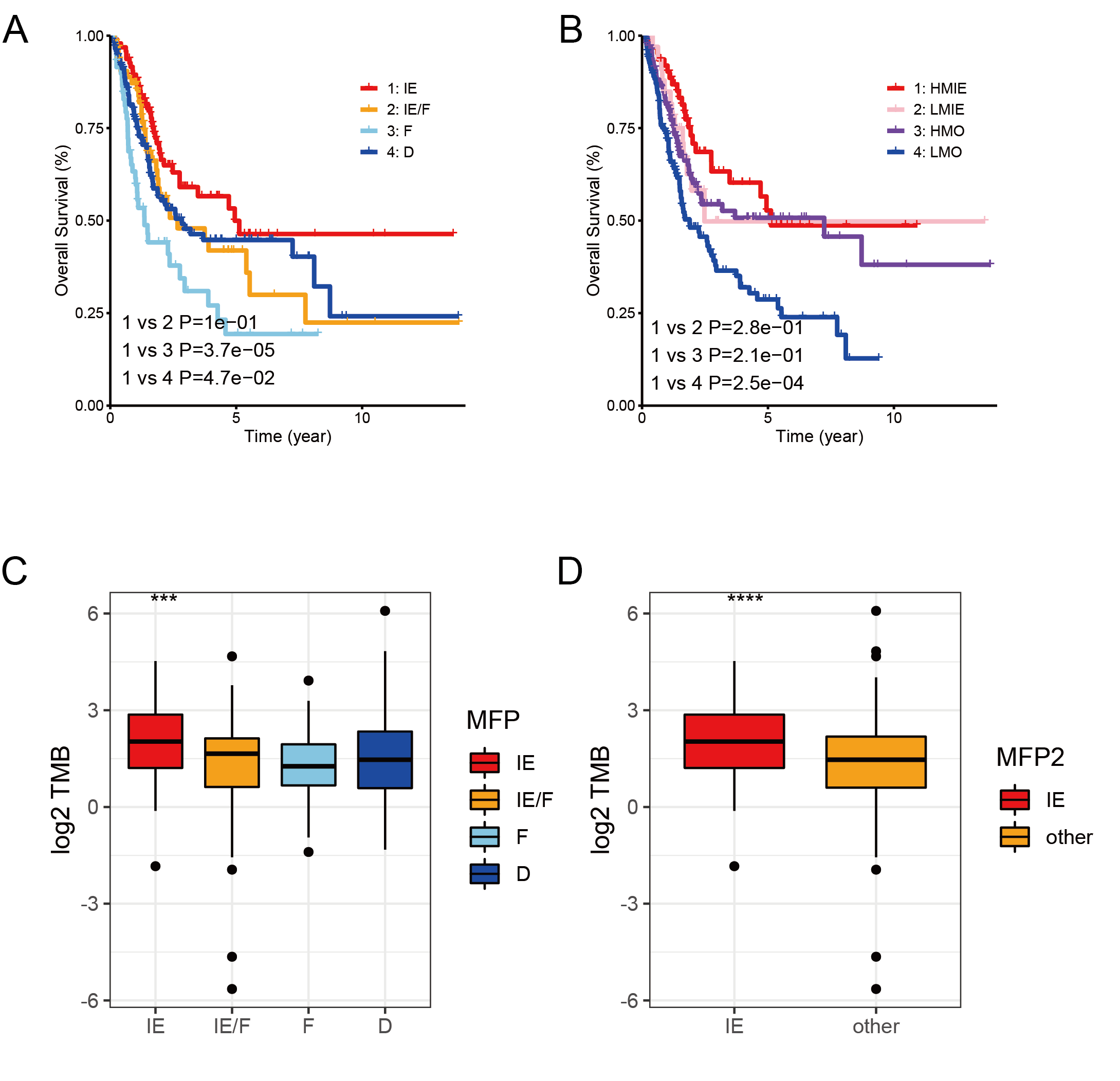

Supplement: Supplementary Figure 1 — Survival analysis based on TME subtypes and TMB patient group. (A) Overall survival of bladder cancer patients stratified by four TME subtype classification on the TCGA-BLCA cohort. (B) Overall survival of bladder cancer patients grouped by the TMB value larger than or smaller than the median of TMB value. Patients with high TMB value have a good survival. (C) Boxplot of log2 transformed TMB value across the four TME subtypes. TMB is the highest in IE subtype. (D) Boxplot of log2 transformed TMB value across the 2 TME subtypes. Subtypes rather than IE were grouped together as other. [file Image_1.tif]

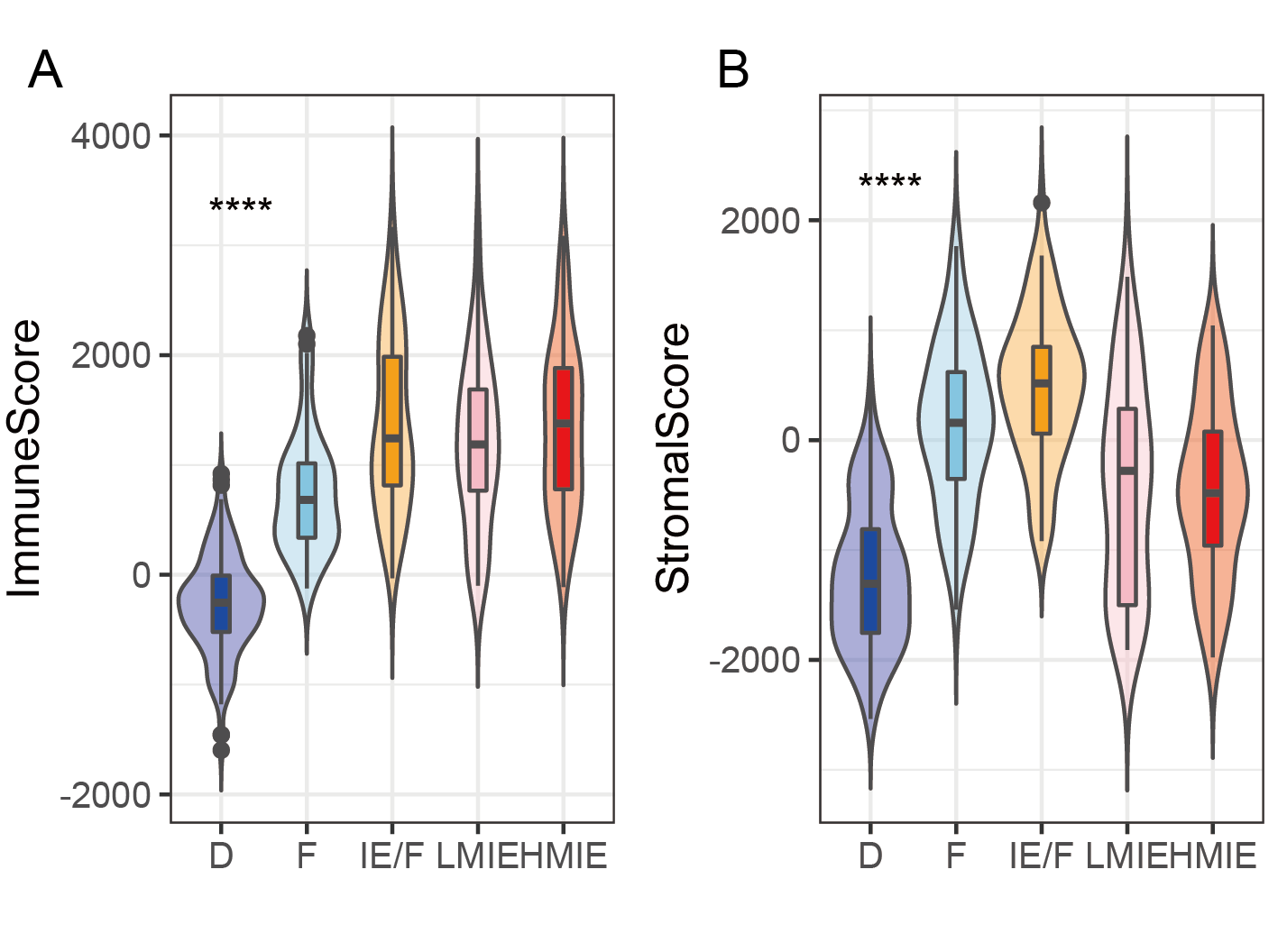

Supplement: Supplementary Figure 2 — Violin plots. (A) The distribution of Immune score between 5 TME subtypes, pvalue were determined by ANOVA-test. (B) The distribution of Stromal score between 5 TME subtypes, pvalue were determined by ANOVA-test. [file Image_2.tif]

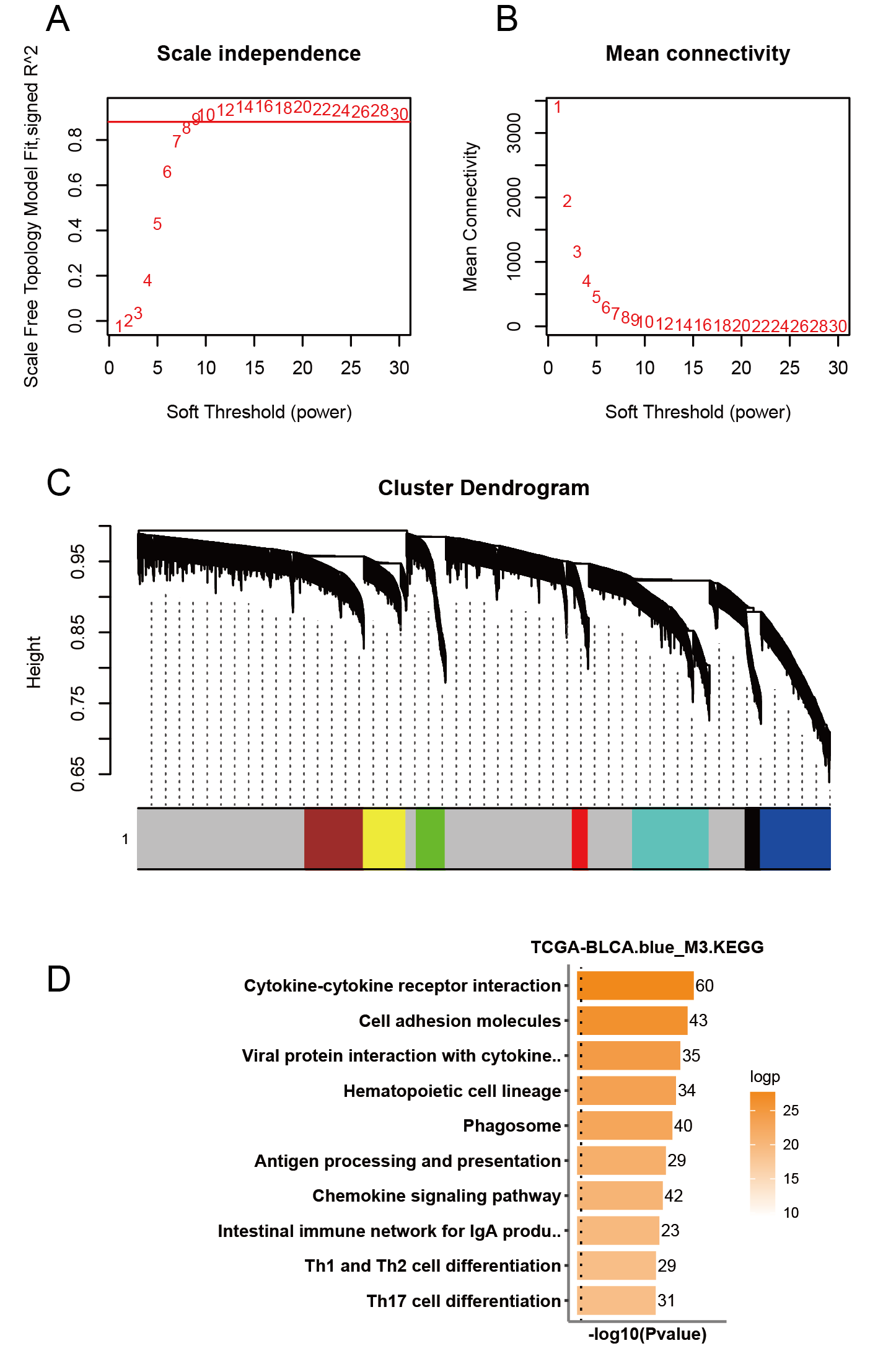

Supplement: Supplementary Figure 3 — WGCNA construction. (A) Determine soft-thresholding power in WGCNA. (B) The scale-free fit index for various soft-thresholding powers (β) (left). The mean connectivity for various soft thresholding powers (right).(C)WGCNA cluster dendrogram on bladder cancer patients, genes were grouped into 7 distinct modules.(D) KEGG pathway enrichment of genes belong to M3 module. [file Image_3.tif]

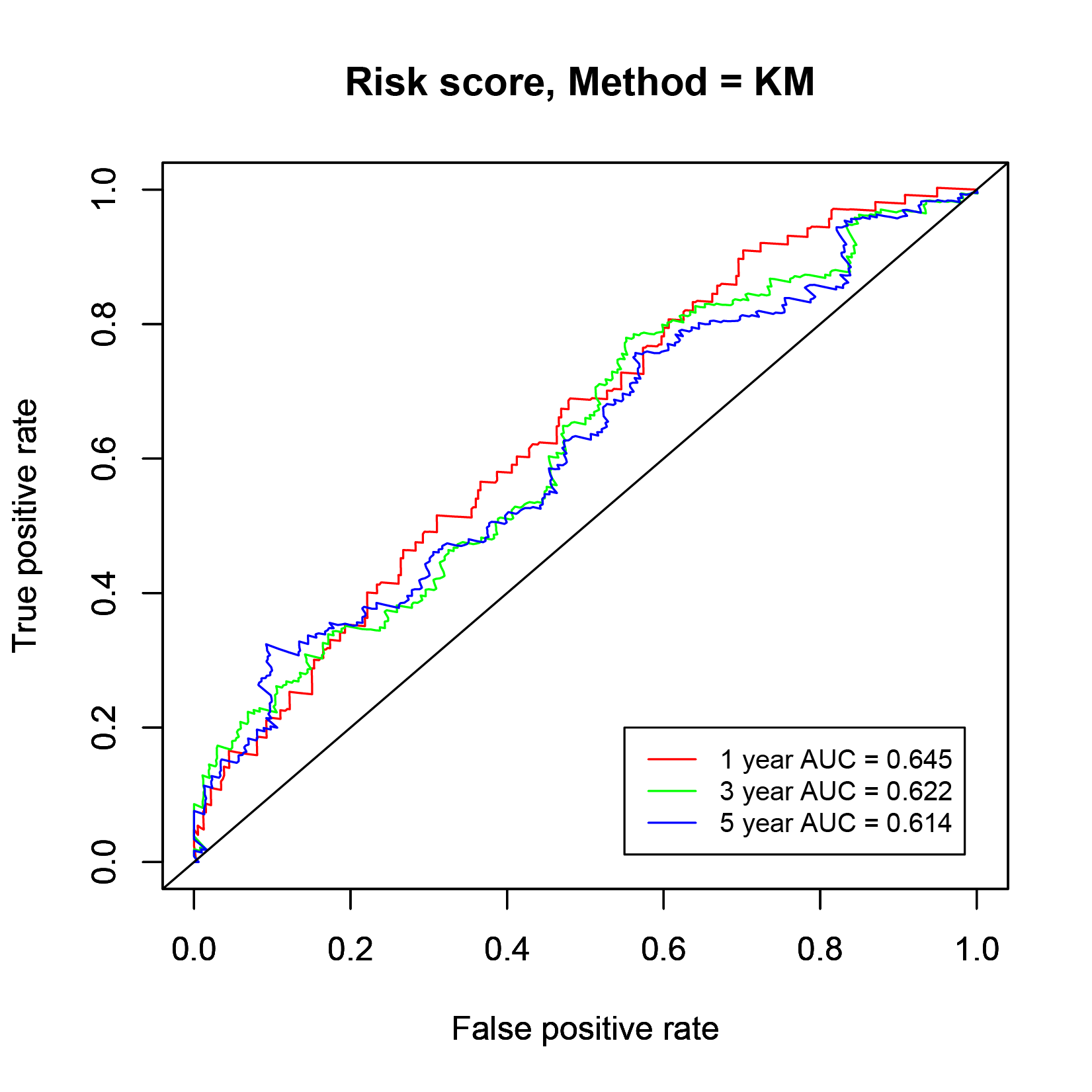

Supplement: Supplementary Figure 4 — ROC curve for lncRNA risk-model. [file Image_4.tif]
