# Supplementary material for: Characterization of the immune related lncRNAs in bladder cancer to aid immunotherapy
Source: Front Immunol. 2022 Aug 26;13:941189. doi: 10.3389/fimmu.2022.941189 (PMC9462669; doi:10.3389/fimmu.2022.941189)
Supplement: Supplementary file 5 [file Table_1.docx]

Table S1 Clinical characteristics of the TCGA-BLCA dataset

| Term | Count | Percent |
| --- | --- | --- |
| All | 403 | 100.00% |
| Gender  Female  male | 105  298 | 26.05%  73.95% |
| Cancer_status  Tumor free  With tumor  Unknown | 231  134  38 | 57.32%  33.25%  9.43% |
| Age  Old  Young | 295  108 | 73.20%  26.80% |
| Grade  High Grade  Low Grade  Unknown | 380  20  3 | 94.29%  4.96%  0.74% |
| Stage  Stage I+II  Stage III+IV  Unknown | 130  271  2 | 32.26%  67.25%  0.50% |
| Tumor_site  Bladder - NOS  Dome  Neck  Trigone  Unknown  Wall Anterior  Wall Lateral  Wall NOS  Wall Posterior | 202  24  7  31  7  25  46  22  39 | 50.12%  5.96%  1.74%  7.69%  1.74%  6.20%  11.41%  5.46%  9.68% |
| Stage_T  T1+T2  T3+T4  Unknown  Stage_N  N+  N0  Unknown | 200  197  6  126  235  42 | 49.63%  48.88%  1.49%  31.27%  58.31%  10.42% |
| Stage_M  M0  M1  Unknown | 195  11  197 | 48.39%  2.73%  48.88% |
| Tissue_prospective_collection_indicator  No  Yes  Tissue_retrospective_collection_indicator  No  Yes  Unknown | 196  207  206  196  1 | 48.64%  51.36%  51.12%  48.64%  0.25% |
| Ethnicity  Hispanic or latino  Not Hispanic or latino  Unknown | 8  363  32 | 1.99%  90.07%  7.94% |
| Other_dx  No  Yes | 296  107 | 73.45%  26.55% |
| History_of_neoadjuvant_treatment  No  Yes | 393  10 | 97.52%  2.48% |
| Vital_status  Alive  Dead | 297  106 | 73.70%  26.30% |
| Diagnosis_subtype  Non-Papillary  Papillary  Unknown | 268  130  5 | 66.50%  32.26%  1.24% |
| Has_new_tumor_events_information  No  Yes | 238  165 | 59.06%  40.94% |
| Has_follow_ups_information  No  Yes | 107  296 | 26.55%  73.45% |
| Has_drugs_information  No  Yes | 270  133 | 67.00%  33.00% |
| Has_radiations_information  No  Yes | 346  57 | 85.86%  14.14% |
